# Reducing Pesticides and Increasing Crop Diversification Offer Ecological and Economic Benefits for Farmers—A Case Study in Cambodian Rice Fields

**DOI:** 10.3390/insects12030267

**Published:** 2021-03-21

**Authors:** Cornelia Sattler, Julian Schrader, Rica Joy Flor, Makarakpakphea Keo, Sokunroth Chhun, Saban Choun, Buyung Asmara Ratna Hadi, Josef Settele

**Affiliations:** 1 Helmholtz Centre for Environmental Research—UFZ, Department of Community Ecology & Department of Conservation Biology and Social-Ecological Systems, Theodor-Lieser-Straße 4, D-06120 Halle, Germany; jschrad@uni-goettingen.de (J.S.); josef.settele@ufz.de (J.S.); 2 Sustainable Impact Platform, International Rice Research Institute, IRRI-Cambodia Office, Phnom Penh 12101, Cambodia; r.flor@irri.org (R.J.F.); buyung.hadi@fao.org (B.A.R.H.); 3 Department of Rice Crop, General Directorate of Agriculture, Sangkat Toeuk Laak 3, Khan Toul Kok, Phnom Penh 12101, Cambodia; keopakphea1993@gmail.com; 4 Royal University of Agriculture, Sangkat Dangkor, Khan Dangkor, Phnom Penh 12101, Cambodia; 5 Provincial Development of Agriculture Forestry and Fisheries Battambang, DomnakLoung Village, WatKor Commune, Battambang 02358, Cambodia; chhunsokunroth@gmail.com; 6 Provincial Development of Agriculture Forestry and Fisheries Prey Veng, Village 4, Kampong Leav Commune, Prey Veng 141303, Cambodia; chounsaban6@gmail.com; 7 Food and Agriculture Organization of the United Nations, Vialle delle Terme di Caracalla, 00153 Rome, Italy; 8 German Centre for Integrative Biodiversity Research (iDiv), Halle-Jena-Leipzig, Deutscher Platz 5e, 04103 Leipzig, Germany; 9 Institute of Biological Sciences, College of Arts and Sciences, University of the Philippines, Los Baños, College, Los Baños 4031, Philippines

**Keywords:** arthropod richness, biological control, bund plants, conventional farming, coupled human and natural systems, ecological engineering, landscape heterogeneity

## Abstract

**Simple Summary:**

Intensified rice cultivation is mostly associated with high input of pesticides. Beneficial arthropods decrease in such environments while pesticide-resistant herbivores can increase, which, in turn, leads to even higher pesticide applications. To break the vicious circle, it is important to implement sustainable farming approaches. Here, we tested such an approach called “ecological engineering” (EE), where non-rice crops were grown in the surroundings of rice fields to provide additional food sources for beneficial arthropods. Farmers did not spray EE fields with pesticides in contrast to conventionally farmed fields, which had no crops in the surroundings, serving as a comparison. Additionally, we included control fields, which were neither treated with pesticides nor had crops in the surroundings. We interviewed farmers to obtain insight about their preference for crops growing in the surroundings and their willingness to use this approach. Our results showed that the yield of EE rice fields was equal to that of conventionally farmed fields. In addition, the benefit–cost ratio was highest for EE and the control fields highlighting their economic advantage. The abundance of parasitoids was lower in conventionally farmed treatments. The proper implementation of EE in combination with farmers’ choice of crops is a promising solution towards sustainable rice production.

**Abstract:**

Rice production is often associated with high pesticide input. To improve farmers’ practice, sustainable management approaches are urgently needed, such as ecological engineering (EE), which aims at enhancing beneficial arthropods while reducing pesticides. Here, we implemented and tested EE in Cambodian rice fields by comparing: (i) fields not treated with pesticides (control); (ii) fields not treated with pesticides but with non-rice crops planted in the surrounding (EE); and (iii) conventionally farmed fields using pesticides (CR). Using benefit-cost analysis, we compared the economic value of each treatment. The non-rice crops preferred by men and women farmers as well as farmers’ willingness to implement EE were assessed using surveys. We sampled arthropod abundance and richness in rice fields and bunds during two seasons. During the dry season, we compared EE and CR among three Cambodian provinces. During the wet season, we specifically assessed the differences in EE, control and CR in arthropod abundance and rice yield in one province. While withholding from using pesticides did not result in a decrease in yield in EE and control treatments, parasitoid abundance was higher in both treatments during the wet season. The benefit–cost ratio was highest for EE and control treatments. Pesticides were likely the main driver causing low arthropod abundance, without any benefit towards increased rice yield. The proper implementation of EE coupled with farmers’ knowledge of ecologically based pest management is a promising solution towards sustainable rice production.

## 1. Introduction

Since the green revolution in the 1960s, rice agroecosystems in Southeast Asia are mostly associated with intensified rice monoculture [1,2]. The trend of intensified rice production and increasing harvested areas is associated with increasing agrochemical inputs like fertilizers and pesticides [3,4,5]. The importance of pesticides has dramatically increased in recent decades since many farmers have increased their pesticide use as they believe it is the only way to prevent pest outbreaks [6,7]. However, pesticides can be harmful not only for the targeted rice pests but also to the environment and human well-being, since pesticides are often the first choice for pest management [8,9,10,11]. To improve the stated situation, habitat management as a form of biological control has increasingly gained interest e.g., [12,13,14,15].

One promising approach to increase landscape diversity and to counteract pesticide inputs is ecological engineering (EE). EE is associated with habitat management [16] and aims to design ecosystems in a sustainable way that benefits both humans and the environment [17]. EE can act as “bottom–up” control in form of habitat manipulation as well as “top–down” control in the form of promoting natural enemies [18]. Habitat manipulation in rice agroecosystems can be implemented by cultivating additional plants on rice bunds, which are earthen mounds surrounding rice fields to keep the water level in the fields [19].

The implementation of EE is based on ecological knowledge [20]. Steps to correctly implement EE in rice agroecosystems include, among others, identifying the principal pest species and the candidate plant species, which should be used for habitat management [21]. The inappropriate selection of plant species can have negative side effects or can be even toxic for natural enemies as plants can contain inhibiting substances like, e.g., xylose, mannose or phenol [22]. Likewise, the selected plants could also have no effect on increasing the abundance of natural enemies [23].

Gurr et al. [24] stated that the receptiveness of farmers to EE will depend on the bund plants that can provide additional income. Aside from several studies on non-crop species, crop species have also been tested as EE components [4]. For example, sesame plants (*Sesamum indicum* L.) were successfully tested as candidate plants in laboratory and field studies in China [25,26,27,28]. Due to the provision of additional food sources, sesame plants improved the survival rate of beneficial insects like parasitoid wasps and predatory mirid bugs. Similar findings by Gurr et al. [29] from a multi-site field study, showed that nectar-producing plants on rice bunds led to an increase in parasitoids and predators. 

EE is a relatively new approach applied to rice agroecosystems in East and Southeast Asia. Field studies that were mainly done in China, the Philippines, Vietnam and Thailand showed contradicting results on the effect of additional plants on rice bunds on arthropod communities, which seems to depend on the selected plants [23,25,29]. However, all studies concurred that the withholding of pesticides enhances biological control in the form of natural enemies in rice fields [23,29,30].

Despite the ecological advantages of bund plants, considerable agronomic constraints have been raised in cultivating additional plants—including labor, seeds, fuel and other capital [16]. This shows how the human dimension is an important factor in the adoption and impacts of EE. This, however, is often neglected in scientific studies on EE. Two aspects are especially essential for the successful implementation of EE: first, the willingness to implement additional plants in the surroundings of rice fields as well as the preference of farmers regarding additional plants need to be tested; second, the ecological impacts of such plants on the ecosystem should be considered, particularly their effects on important insect functional groups like predators, parasitoids, detritivores and herbivores within the rice fields. Studies on both the economic benefit–cost ratio as well as the ecological effects of habitat manipulation are limited, which warrants further research [31,32].

Here, we implemented an approach that considers both human and natural systems. Through a farmer participatory method (gender-informed survey and experiments in the fields of farmers), we tested crops potentially suitable to use for EE in rice fields. We considered that following the preferences of farmers in a farmer participatory experiment covers aspects such as skill, tools and labor for cultivation as well as seed sources. At the same time, involving farmers in our field experiments helped to gain information on plants that are both preferred by farmers and can be used for EE. Crops cultivated on the bunds should be familiar and have perceived benefits for farmers to create incentives to use the approach of EE in the future.

These could help to address the agronomic constraints for EE. At the same time, we tested the effect of crops selected by farmers, on arthropod natural enemies and herbivores in rice fields as well as the effect on insect family richness. Furthermore, we estimated the socio-economic effects of EE by performing partial benefit–cost calculations, focusing on paid-out costs, which are a key consideration for smallholder farmers.

## 2. Materials and Methods

Our experiment took place over two consecutive seasons in 2019 in Cambodia. Cultivation is strongly influenced by monsoon seasons. Two distinct seasons occur: the dry season, which is from November to April; and the wet season, which is from May to October [33]. The mean monthly rainfall ranges from 14.7 mm in January to 321.1 mm in September. The mean monthly temperature ranges from 25.7 °C in January to 29.4 °C in April (mean rainfall and temperature from 1991–2015 [34]). Due to an El Nino event in 2019 [35], the start of the wet season varied among the different provinces. Therefore, we focused on one province only during the wet season to prevent high variances. In the selected province, farmers started cultivating rice in June while, under average conditions, farmers would start in April to coincide with the first rainfalls.

### 2.1. Study Design

Dry season: in the dry season, we selected a total of ten rice fields in three provinces: the first province, Prey Veng, is situated 80 km east of the capital Phnom Penh. Rice fields were selected close to Sdao village (11°25′ N 105°20′ E). The second province, Kampong Thom, was ca. 150 km north of Phnom Penh. The rice fields were located within Balang area (12°41′ N 104°53′ E). The third province, Battambang, is 300 km northwest of Phnom Penh (13°05′ N 102°59′ E). In all three provinces, rice constitutes the predominant crop and is intensively cultivated.

**Figure 1:**
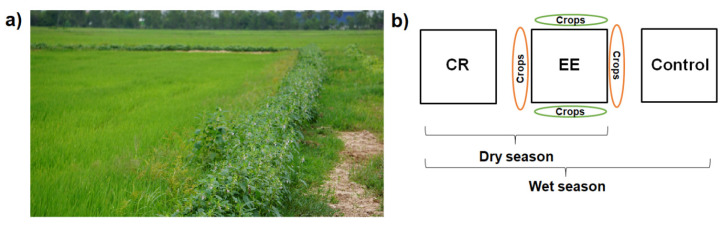
(**a**) Example of one ecologically engineered rice field (EE); and (**b**) the study design during the dry and wet season. Conventionally farmed rice fields (CR) were treated with pesticides, EE fields were not treated with pesticides and cultivated with additional plants on the rice bunds (earthen mound between rice fields to keep water level in the fields) and fields with no pesticides application and no bund plants served as control fields.

In each province, we selected two pairs of rice fields, respectively, except in Kampong Thom province, where we only selected one pair of rice fields due to logistical reasons. One pair of rice fields consisted of one field with ecological engineering (EE) and one conventionally farmed rice (CR) field (Figure 1). EE rice fields were cultivated with mung bean (*Vigna radiata* (L.) R. Wilczek) and sesame (*Sesamum indicum* L.) plants in the surroundings of the rice fields on rice bunds, which are earthen mounds between rice fields to keep the water level in the fields. Both plants were already successfully cultivated as bund plants in recent studies in the Philippines and China [20,25]. For mung bean plants, we used *CARDI chey* mung bean seeds, which is a commonly planted variety developed by the Cambodian Research and Development Institute [36]. For sesame plants, we used seeds from local markets that are also commonly used by farmers. EE rice fields were embedded within four bunds of which we planted two bunds with mung beans and two with sesame. Bund plants were planted in two rows next to the rice fields on bunds that were accessible to farmers without destroying the bund crops. In EE fields, neither the crops on the rice bunds nor the rice fields were treated with pesticides. Exceptions were the rice fields in Battambang province where weed pressure is relatively high [37] and farmers were afraid of high harvest loss. In these fields, herbicides were applied during the seeding and tillering stage of rice plants. CR fields were treated with pesticides and had no additional plants in their surroundings. To obtain information about the pesticide use in CR fields, farmers were asked about the frequency and type of pesticide they used before arthropod sampling. The number of pesticide applications ranged from one to three applications with any combination of insecticides and herbicides, which is a common practice in these provinces [7]. Farmers in Prey Veng and Kampong Thom provinces cultivate common rice varieties like *IR504*, which only takes around 90 days from seeding to harvest. In Battambang province, farmers cultivate *Srov Ngor* rice variety, which has a growing duration of around 120 days. Rice farmers apply broadcast seeding, with high seed rates, resulting in high density of rice plants [10]. The rice fields of each pair were at least 100 m apart from each other to avoid interactions. The size of the rice fields ranged from 1200 to 8000 m^2^. In total, we investigated five rice pairs in the dry season whereas the field condition of pairs was equated.

Wet season: due to an El Nino event, rainfall varied considerably during the wet season in 2019 [35]. To avoid high rainfall variability between different provinces, we focused only on the Prey Veng province during the wet season. To distinguish between the effects of pesticides and bund plants on the functional groups of the arthropod community, we expanded our study design from two to three treatments for comparison (Figure 1b) during the wet season. The third treatment served as a negative control where neither pesticides were applied nor crops on the surrounding bunds were planted. The field size ranged from 3000 to 8000 m^2^ whereas the field condition of trios was equated.

In total, five trios were selected (15 rice fields in total). Similarly to the dry season, trios were at least 100 m apart from each other and the number of pesticide applications in the CR fields were determined by farmers who sprayed only once per field with either herbicides or insecticides during our experiment. Farmers cultivated the rice variety *OM5451,* which takes 90 days, similarly to *IR504*.

### 2.2. Gender-Informed Survey and Bund Plants

To identify additional plants with potential for inclusion in the EE experiments during the wet season, we conducted a gender-informed survey before the experiment was implemented. We separately interviewed female and male farmers in Prey Veng province, with the aim of capturing knowledge domains covered by both genders. A total of 30 female and 30 male farmers (*n* = 60) were randomly selected in two sites in Prey Veng province, where the experiments were conducted. The questionnaire for male farmers included rice cropping practices as they are commonly managing this within the household. The questionnaire for female farmers focused on both crops cultivated and collected (see QS1 and QS2 for details). The respondents identified crops that they cultivated, as well as those found useful near their homes, and around their farms. To aid recall and collect samples for identification, the interviews were combined with a transect walk around their farms [38]. Analysis was done on species cultivated and collected, comparing between men and women farmers. The count of unique species identified per farmer was done. Then, a one-way ANOVA was performed to see whether there are differences in number of species collected or cultivated between men and women.

Farmers were then asked to rank the top plants they considered beneficial. At the same time, 30 farmers interviewed on their rice cultivation were asked about their willingness to plant crops in the surroundings of their rice fields.

As not all plants preferred by the farmers qualified for use in the EE experiments, we selected only those plants that were currently cultivated in the province and which met the following criteria: (1) short growing duration; (2) flowers in time with the rice growing period; and (3) could grow on rice bunds. Plant species ranked by farmers included both non-crop and crop species. The top two cultivated crops, which met the above stated criteria for EE, were selected for the experiment and cultivated on the bunds during wet season. These were sponge gourd (*Luffa aegyptiaca* Mill.) and chili (*Capsicum annuum* L.), which were planted on the rice bunds together with mung bean and sesame plants. Crops were planted separately in rows, so that each bund was cultivated with only one of the crops. However, during our field experiment, the chili plants did not survive on the rice bunds and were replaced with additional plants of sponge gourd. Similarly to the dry season, crops were planted in two rows next to the rice fields.

### 2.3. Benefit–Cost Ratio of Treatments

During the wet season we recorded for each treatment the costs per hectare. These included labor for pest management, land preparation and harvest, and input costs for fertilizer, pesticides and seeds incurred. Rice yield was assessed using crop cut data from randomly selected 1m × 1m squares in each treatment. Yield from the bund crops, harvested as they matured, were provided by the farmers involved in the experiment. Costs for the labor of rice land preparation, seeds and crop harvesting were held constant across all treatments, using proxy values from a 2016 survey data of rice farmers in the same province [10]. Based on this, we computed a benefit–cost ratio using a partial benefit–cost analysis. Only paid-out costs were considered, which excluded the labor costs of the farmer or farm household.

### 2.4. Arthropod Sampling and Analysis

Arthropods were sampled on one occasion during the booting stage of the rice plants using sweep nets (32 cm in diameter, 68 cm depth, <1 mm mesh size). In each rice field, we collected four sample units by walking with a speed of approximately 0.5 m/s within the rice fields while conducting ten sweeps, respectively. To broaden our knowledge about arthropods occurring in the cultivated crops, we additionally sampled them by walking transect lines on the bunds and conducting ten sweeps per bund (in total four bunds = four sample units). These samples were not used for statistical analysis. Arthropod communities in crops on rice bunds and rice plants were sampled separately at the same date. Arthropods were counted, identified at the family level and assigned to functional groups, which are based on a similar functional behavior and food acquisition strategies. Arthropods were assigned to their feeding guild in rice agroecosystems based on Shepard et al. [39,40] and Heong et al. [41] and categorized into the following functional groups (Table S1):

- Predators: consist of all predatory arthropods that actively or passively hunt for other arthropods. This includes, among others, active and passive hunting spiders, predatory bugs, flies and beetles.
- Parasitoids: all endo- and ectoparasitoids were assigned to the functional group of parasitoids. Parasitoids consist mostly of hymenopteran wasp as well as flies and Strepsipterans (twisted-wing parasites).
- Herbivores: arthropods feeding on rice plants as sap sucker, defoliators, miners, root feeders or stem borers were categorized as herbivores.
- Detritivores: arthropods consuming dead organic material were assigned to the functional groups of detritivores.
- Pollinators: flower-visiting arthropods, which in our case refers to the plants growing on the bunds.

Based on the literature, the abundance and important functionality, we selected ten main families for further analysis. Subsequently, ten main families were identified and analyzed: Araneidae (predator), Chironomidae (detritivore), Cicadellidae (herbivore), Coccinellidae (predator), Delphacidae (herbivore), Entomobryidae (detritivore), Mymaridae (parasitoid), Tetragnathidae (predator), Thripidae (herbivore), and Trichogrammatidae (parasitoid) [41,42,43].

To test the effect of the different treatments on the abundance of functional groups as well as the identified main families in rice fields, we used generalized linear mixed effect models with negative binomial distribution, as abundance data were overdispersed. Data of dry and wet season were separately analyzed and sample units (four per field) were merged. In both models, we included an interaction effect of treatments (EE, CR, and control only in wet season) with functional groups. To control for possible variation among regions and pairs, we included pairs nested in regions as a random effect in the dry season model. For the wet season, we included trios as a random effect only because all trios were located in the same region. We used a post-hoc pairwise test (Tukey test) based on our computed models to compare pairwise the functional group abundance and main families within the treatments. In addition to the abundance comparison of functional groups and main families, we also compared the family richness (number of families per treatment) and relative abundance of functional groups among pairs and trios.

## 3. Results

### 3.1. Gender-Informed Survey and Bund Plants

In total, the surveyed farmers identified 197 different species of plants that were cultivated or collected in the farm environment. Male farmers mentioned on average 18 plant species that they cultivated, while the female farmers mentioned 19 species. The top selected crops by farmers can be found in Table 1. There was no statistically significant difference between males and females in the crops cultivated. Notably, the men identified five species on average (SD 3.3) that they collected near the rice farms, while the women identified four species (SD 2.7). There is a statistical difference between the two groups (*p* < 0.05) in a number of crops collected near the rice field. Farmers’ willingness to cultivate crops in the surroundings of their field was rather low. Only 7% of interviewed farmers were willing to plant vegetables on the bunds. All surveyed famers, however, cultivated non-rice crops near their farms or households.

We found many parasitoids and predators of rice pest in our tested bund plants: mung bean, sesame and sponge gourd collected by sweep netting (>25%; Table S2). Herbivores (1178 specimens) were the most abundant functional group collected with sweep netting, followed by detritivores (1013), predators (432) and parasitoids (319). Herbivores were mainly composed of aphids (440), which we rarely collect inside rice fields, followed by Thysanoptera (342) and Cicadellidae (108). Predators found on bund plants were dominated by spiders (233) and the predatory mirid bug (57), which also occur in rice fields as natural enemies.

### 3.2. Benefit–Cost Ratio of Treatments

During the dry season, rice yield in EE fields was on average 4426 kg/ha ranging from 3775 to 4800 kg/ha. In CR fields, the rice yield was on average 4353 kg/ha ranging from 3641 to 5500 kg/ha. During the wet season, the rice yield was on average 6100 kg per ha in EE fields (ranging from 5000 to 7025 kg/ha), 6235 kg/ha in control fields (5950 to 6500 kg/ha) and 6075 kg/ha (4650 to 6325 kg/ha) in CR fields (Table 2). On average, the mung bean, sponge gourd and sesame plants yielded 17.6, 20.5 and 9.5 kg per EE field (calculated per hectare), respectively. Partial cost benefit analysis conducted during wet season, showed that the highest cost incurred in CR fields, due to high labor costs of pesticide and pest management. The lowest cost incurred in the control fields ranging from USD 656.61 to USD 718.85 per ha (mean USD 693.03). The benefit–cost ratios were similar for the control and EE (ratio of 2.02), followed by CR (1.84) fields. A summary of all costs and revenues per treatment can be found in Table 2. This calculation did not include the costs for chili plants within EE fields. There is a slight difference in total costs when costs for chili (no yield during the experiment) are included; the mean benefit–cost ratio for EE is lower (2.00), which is still higher than CR.

### 3.3. Arthropod Abundance and Richness during Dry Season

Overall, we collected 16,255 specimens of arthropods from the rice fields. The most abundant functional group were detritivores (8446 specimens), followed by herbivores (3894), parasitoids (2136), predators (1777) and pollinators (2).

Chironomidae, which belong to the group of detritivores, were the most abundantly collected insect family in CR and EE treatments, followed by Trichogrammatidae, which belonged to the group of parasitoids (Table S1, Figure S1).

Generalized linear mixed effect model showed no significant differences of functional group abundance between treatments (Table 3). This was similar found for the main families analyzed (Table S3).

**Figure 2:**
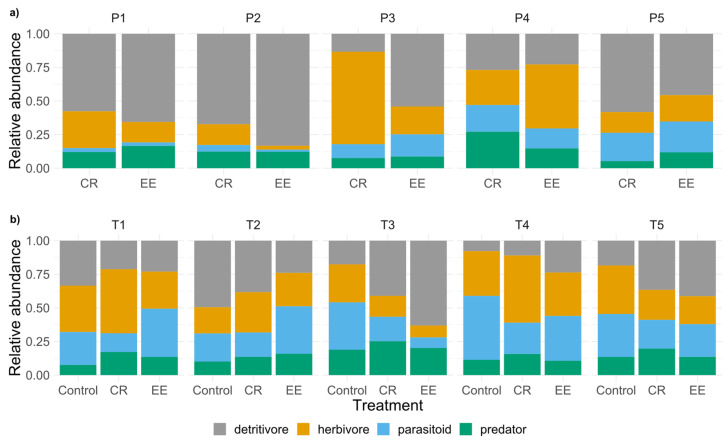
Relative abundance of the functional groups per rice field collected in different treatments (CR = conventional rice field; EE = ecologically engineered field; control). Rice fields were compared in pairs (P1–P5) during the (**a**) dry season and compared in trios (T1–T5) during the (**b**) wet season.

The relative abundance of functional groups highly varied among the rice field pairs (Figure 2a). Predators were slightly higher in EE rice fields except for one rice field pair where we found more predators in the CR field. In three out of five fields, we found a lower abundance of herbivores in EE fields. In the same three out of five fields, we found higher detritivores in the EE fields. Parasitoids showed no trend in their relative abundance and varied among the treatments.

The family richness of rice field pairs was always higher in EE rice fields and ranged from a maximum of 62 families in EE fields to 26 families in CR fields (Table S4).

### 3.4. Arthropod Abundance and Richness during Wet Season

In total, we sampled 30,049 arthropod specimens during the wet season in the rice fields. Detritivores were the most abundant group (8910 specimens), followed by herbivores (8571), parasitoids (8222), predators (4345) and pollinators (1). Generally, the insect family of Trichogrammatidae was found in the highest numbers in control and EE treatments (Figure S1b), followed by Chironomidae and Thripidae. In CR fields, Chironomidae were the most abundant family (Table S1).

Functional group abundance of parasitoids was significantly higher in EE (*p* < 0.05) and control (*p* < 0.01) than in CR treatments (Table 3). The family of Trichogrammatidae was significantly more abundant in EE treatments (*p* < 0.05) than in CR treatments. This was similarly found for the family of Entomobryidae, which were significantly higher in EE (*p* < 0.001) and control (*p* < 0.01) compared to CR treatments. Delphacidae were found in lower numbers in controls compared to CR treatments (*p* < 0.01). Contrary to this, Thripidae were found in higher numbers in control than in CR treatments (*p* < 0.05) (Table S3).

Similarly to the dry season, relative abundance of functional groups of field trios highly varied among the treatments (Figure 2b). We found the highest relative abundance of parasitoids in two out of five EE treatments. Predators were found to be slightly higher in CR fields than in EE and control fields. Whereas herbivores were found in the lowest abundance in EE fields, detritivores varied among the treatments and showed no trend.

In contrast to the dry season, family richness highly varied among the treatments. In three out of five trios, control treatments contained highest family richness. We found higher family richness in CR fields compared to EE treatments within three trios and only in one trio did we find the highest family richness in EE treatments (Figure 2b).

## 4. Discussion

Here, we highlighted the multifaceted opportunities offered by the approach of ecological engineering in Cambodian rice fields. Rice fields treated by ecological engineering provided additional income and yield for farmers by crops cultivated on rice bunds and resulted in an equally high yield compared to conventionally farmed fields despite withholding pesticides. Our results further indicated that especially the abundance of parasitoids—an important insect group for pest management—was positively affected by withholding from using pesticides. All this considered together indicates that ecological engineering can lead to (i) enhanced food provisioning for farmers while; (ii) decreasing costs; and to (iii) positive effects of pesticide withholding on important functional groups.

### 4.1. Gender-Informed Survey and Bund Plants

EE can promote crop diversification, which in turn, provides more diverse food supplies for farmers [23]. We intentionally integrated farmers’ crop preferences to our study design. This mostly included vegetable crops that farmers already grew elsewhere on their land. While most plants seemed well suited to grow on rice bunds (such as mung bean, sponge gourd, sesame), some crops selected by farmers did not grew well here (e.g., chili). This is possibly because these plants were not adapted to the often water-saturated soil of rice bunds. This highlights a caveat in the farmers’ choice to implement EE. The best additional crop plant needs to match both the preferences of farmers and the ecological conditions of the rice bunds.

Another challenge to implement EE in Cambodian rice fields was connected to social aspects: farmers build farm management skills with both environmental learning (assessing trade-offs from their practice) and social learning (decisions based on imitating others) [44,45]. In a situation where farmers are bombarded with information on pesticides, and neighboring farmers are using pesticides [10], there is greater dependence on social learning [46]. Presently, the social context would influence decisions towards pesticide use. It would therefore not be enough to assume that when farmers obtain benefits from the additional bund plants, they would automatically practice the technique in the future. This tendency was also mirrored during our survey in the wet season where most of the famers stated to be not willing to cultivate crops on their bunds. Farmers require more time and support to enable skilling where they experiment on how to work with these diverse crops together and observe the outcomes [47].

Enabling environmental learning, as well as providing a balance for social learning by implementing at larger scale would be useful for introducing EE in farming communities.

### 4.2. Benefit–Cost Ratio of Treatments

When comparing the benefit–cost ratio among the rice field treatments, EE and the control performed better than the CR farmed fields. Zou et al. [48] questioned the chemical-based pest management in a sub-tropical rice agroecosystem in China, as only in less than half of their studied cases pesticides were profitable. They found no dependencies between the biocontrol and pest damage with landscape composition. Different to our study, they found 20% higher yield in pesticide-treated plots than in untreated plots. In our study, the costs were highest in CR fields, which also had lower parasitoid abundance in the wet season possibly due to pesticide application. Even though herbivore abundance was similarly high among treatments, rice yield was comparable between treatments and did not decrease by withholding pesticides. This might be because the abundance of major rice pests like the Brown Planthopper (*Nilaparvata lugens*, STÅL) and the White Backed Planthopper (*Sogatella furcifera*, HORVÁTH) was low during both the wet (<0.1%) and dry seasons (<0.1%).

### 4.3. Arthropod Abundance and Richness

We found significantly higher parasitoid abundance during the wet season in EE and control treatments compared to CR treatments, which indicated that pesticides might be the main cause decreasing parasitoid abundance. Contrary to our expectation, the provisioning of additional nectar sources did not result in significant differences in natural enemy abundance compared to control treatments (no bund plants, no pesticides) in rice fields. This was similarly found by a study of Horgan et al. [23] who found no promoting effect of bean plants growing on bunds for parasitoids and predators. Furthermore, pesticides seemed to be the main driver for negative impacts on pest management. Thus, Horgan et al. [23] suggested that growing crops on bunds might be a good incentive for farmers to avoid pesticide use. In our study, mung bean, sesame and sponge gourd grew well on the bunds and resulted in additional yield. We collected many natural enemies of rice pests like parasitoids and predators (>25%, Table S2) in bund crops. Therefore, sesame, sponge gourd and mung bean seem to be suitable plants for EE in our study region.

Zhu et al. [25,26,27] showed that sesame plants provided important food resources to insect natural enemies and are suitable for implementation in rice agroecosystem in subtropical China. While Zhu et al. [25] indicated a positive relationship between sesame plants and natural enemy abundance, in our study pesticide application might be the major driver for reduced parasitoid abundance (see also [23]). However, crop heterogeneity can be an important driver for multi-trophic diversity [49,50]. In addition to biological control, other ecosystem services like pollination can be promoted by EE [51]. On our selected bund plants, we found pollinating flies that can also act as a predator in rice fields. Increasing community stability and functioning can prevent pest outbreaks due to the coupled increase in specialist and generalist predators [52]. At the same time, interconnecting bunds and increasing number of rice patches can lead to lower herbivore abundance [53].

When comparing single insect families among our treatments, we found more parasitoids like Trichogrammatidae in EE and control fields than in CR fields during the wet season. This again suggests that pesticides might be the main driver for reduced parasitoid abundance in rice fields (see also [54). Similarly, important taxa of detritivores, like Entomobryidae, were found in fewer numbers in CR treatments compared to EE and control treatments. Detritivores play an important role in rice fields as they can serve as a food source for early arriving predators and can led to an increase in their abundance before herbivores immigrate into rice fields [30].

Family richness was higher in EE compared to CR fields during the dry season, whereas we found no clear trend among the treatments during the wet season. This might indicate that during the dry season, bund plants and the withholding of pesticides might have higher effects on family richness than during the wet season. At the same time, farmers applied pesticides once to twice more often during the dry season than during the wet season. This might further amplify the lower family richness in CR compared to EE fields between dry and wet season. Our results mirror this assumption. We found a lower number of families in CR treatments collected during the dry season than during the wet season.

In the face of climate change, weather conditions may become increasingly unfavorable for insects due to higher temperature or heavy rain fall [31]. EE may, at least partly, counteract the negative effects of climate change on crop yield. For instance, additional crops provide more shelter for natural enemies during heat and heavy rain events [31].

## 5. Conclusions

Mung bean, sesame and sponge gourd are suitable plants to grow on rice bunds and offer multiple opportunities for farmers to diversify and manage pests without relying on pesticides. In our study, farmers gained additional yield by growing crops on bunds and reduced cost by withholding pesticides. Parasitoid abundance was higher in fields without pesticide treatments during the wet season, which indicated that pesticides might be the main driver for a reduction in parasitoid abundance. We found higher family richness in EE fields than in conventional farmed fields during the dry season. Although we found no clear trend of increasing natural enemy abundance by bund crops, EE seems to be a good incentive for farmers to prevent pesticide use and gain additional income.

To increase incentives for farmers, the selection of surrounding plants should integrate information about farmers’ choice to increase potential adoption by farmers. The proper implementation of EE in combination with farmers’ choice of crops is a promising solution towards sustainable rice production. As a next step, the approach of EE should be implemented at the landscape scale with the farmers’ choice of bund plants to increase (i) the resources for insect natural enemies which might show significant effects; and (ii) to increase the participation of farmers in the EE approach.

## Figures and Tables

**Table 1:** Top selected crops by surveyed famers (*n* = 60).

Rank	Common Name	Scientific Name	*n*	% of Respondents
1	Rice	*Oryza sativa*	59	98.3
2	Sponge gourd	*Luffa aegyptiaca*	54	90.0
3	Lemon grass	*Cymbopogon* sp.	49	81.7
4	Banana	*Musa* *acuminata*	48	80.0
5	Papaya	*Carica papaya*	46	76.7
6	Greater galangal	*Alpinia galanga*	40	66.7
7	Mango	*Mangifera indica*	39	65.0
7	Chili	*Capsicum annuum*	39	65.0
9	Coconut	*Cocos nucifera*	38	63.3
9	Turmeric	*Curcuma longa*	38	63.3
11	Bottle gourd	*Lagenaria siceraria*	32	53.3
11	Chinese basil	*Ocimum basilicum*	32	53.3
13	Sweetsop/sugar apple	*Annona squamosa*	30	50.0
13	Sugar palm tree	*Borassus flabellifer*	30	50.0

**Table 2:** Partial benefit–cost analysis (in USD ha^−1^). To compute the benefit–cost ratio (BCR) only paid-out costs were considered, which excluded the labor costs of the farmer or farm household.

	Ecological Engineering (*n* = 5)				Control (*n* = 5)				Conventional (*n* = 5)			
	Min	Max	Mean	SD	Min	Max	Mean	SD	Min	Max	Mean	SD
Land preparation (constant)	459.2	459.2	459.20	0.00	459.2	459.2	459.20	0.00	459.2	459.2	459.20	0.00
Seed cost (rice)	115.0	115.0	115.00	0.00	115.0	115.0	115.00	0.00	115.0	115.0	115.00	0.00
Seed cost (crop)	2.0	2.0	2.0	0.00								
Labor	25.0	25.0	25.00	0.00	0.0	25.0	12.00	12.55	0.0	25.0	5.00	11.18
Fertilizer	19.01	83.00	46.35	24.48	19.01	56.25	43.43	16.51	19.01	78.75	53.18	21.67
Pesticide	0.0	0.0	0.0	0.00	0.0	0.0	0.0	0.00	11.75	46.00	31.25	17.63
Pest mgmt. labor	0.0	0.0	0.0	0.00	0.0	0.0	0.0	0.00	3.0	25.0	15.20	11.32
Harvest (constant)	63.4	63.4	63.40	0.00	63.4	63.4	63.40	0.00	63.4	63.4	63.40	0.00
Total cost	683.61	747.60	710.95	24.48	656.61	718.85	693.03	27.57	672.11	787.35	742.23	48.47
Revenue rice	1125.00	1580.63	1372.50	164.86	1338.75	1490.63	1402.88	68.73	1046.25	1603.13	1366.88	202.54
Revenue mung bean	2.50	76.67	35.17	25.74								
Revenue sponge gourd	1.56	6.67	5.12	1.88								
Revenue sesame	4.69	54.17	23.84	17.86								
Total revenue	1133.75	1718.14	1436.63	153.79	1338.75	1490.63	1402.88	68.73	1046.25	1603.13	1366.88	202.54
**BCR**	1.66	2.30	**2.02**	0.19	1.94	2.09	**2.02**	0.06	1.56	2.07	**1.84**	0.19

*n* = number of rice fields.

**Table 3:** Pairwise comparison of functional groups (Tukey method) based on our computed models of functional group abundance collected in different treatments (CR = conventional rice field; EE = ecologically engineered field; control) during the dry (DS) and wet season (WS).

Season	Functional Group	Comparison	Estimate	SE	*p* Value
DS	Detritivore	CR–EE	−0.406	0.440	0.356
	Herbivore	CR–EE	0.067	0.444	0.880
	Parasitoid	CR–EE	−0.386	0.439	0.380
	Predator	CR–EE	−0.418	0.439	0.341
WS	Detritivore	Control–CR	0.065	0.275	0.970
		Control–EE	−0.359	0.273	0.387
		CR–EE	−0.424	0.274	0.270
	Herbivore	Control–CR	0.245	0.276	0.649
		Control–EE	0.295	0.274	0.529
		CR–EE	0.050	0.274	0.982
	Parasitoid	Control–CR	0.821	0.274	<0.01
		Control–EE	0.187	0.273	0.773
		CR–EE	−0.634	0.274	<0.05
	Predator	Control–CR	−0.084	0.275	0.950
		Control–EE	−0.185	0.274	0.778
		CR–EE	−0.101	0.275	0.928

## Data Availability

The data presented in this study are available in the supplementary material.

## Supplementary Materials

The following are available online at https://www.mdpi.com/2075-4450/12/3/267/s1, Questionnaires (QS1, QS2), a list of collected insects (Tables S1 and S2) as well as statistical models (Tables S3 and S4) can be found in the Supplementary Materials.
