# Supplementary material for: Reducing Pesticides and Increasing Crop Diversification Offer Ecological and Economic Benefits for Farmers—A Case Study in Cambodian Rice Fields
Source: Insects. 2021 Mar 21;12(3):267. doi: 10.3390/insects12030267 (PMC8004109; doi:10.3390/insects12030267)
Supplement: Supplementary file 1 [file insects-12-00267-s001.pdf]

Supplementary Material

Reducing Pesticides and Increasing Crop Diversification Offer Ecological and Economic Benefits for Farmers—A Case Study in Cambodian Rice Fields

Insects

Cornelia Sattler, Julian Schrader, Rica Joy Flor, Makarakpakphea Keo, Sokunroth Chhun, Saban Choun, Buyung Hadi, Josef Settele

Corresponding author: Cornelia Sattler

Co.sattler@gmail.com

UFZ - Helmholtz Centre for Environmental Research,

Department of Community Ecology,

Theodor-Lieser-Straße 4,

D-06120 Halle, Germany

Supplementary Questionnaire S1

Q.No:

Questionnaire

Interview with Female Farmer

I would like to ask you some questions about use of plants and animals in the farm neighborhood. This is for a study to find out how to help farmers grow beneficial plants but are also useful to manage insects.
The objectives of this interview are:
To know what plants are currently used in the farmer field and surrounding agricultural areas.
To know what other benefits are derived from the farm neighborhood.
This interview may take 30 mins-1 hour. The data will be used for research purposes only. Will you consent to be interviewed?

Consent of respondent	Yes □1 No □2
Name of Interviewer:	
Date of interview	

I. Farmer information

1. What is famer name? ____________________Age_________
2. Address: ____________/__________/____________/___________
   Province: a. Prey Veng, b. Takeo
   District: a. Paem Ror, b. Preah Sdach, c. Bati, d. Tran
   Commune: a. Prey Kandeing, b. Lvear, c. Put Sar, d. Sam Bour
   Village: a. Sdao, b. Thom, c. Kandaul, d. Ro Vieng
3. What is your role in the family? Head of household □1
   Wife □2
   Children □3
   Extended family□4
4. Is rice farming your main occupation? yes □1 No □2
5. Aside from rice farming, what are your other income generating activities?
   ____
6. Are you active or significantly involved in farming? yes □1 No □2

II. Making use of plants and animals in the agriculture area.

7. What crops do you grow, in what time of the year, and for what purpose?

Crops	Jan	Fe b	Mar	Apr	May	Ju n	Jul	Au g	Se p	Oc t	Nov	De c	Purpose	Code
														1= Eating<br>2= Sell<br>3=Traditio nal products<br>4= Medicinal plant<br>5=Religiou s purpose<br>6=Livestoc k fodder<br>7= Others

8. What plants growing in the area, which you collect or use, but do not plant yourself? Why do you collect these?

**No**	**Name**	**Purpose**	**When and How many times you collect**

Code: 1 = household consumption, 2 = selling, 3 = traditional products, 4 = medicinal plant, 5 = religious purpose, 6 = livestock fodder, 7 = others

15.1. Of the plants you identified can you name the most important?

9. What animals do farmers collect from the farm and surrounding area? Why do you collect these?

No	Name	Purpose	When and How many times you collect

Code: 1 = household consumption, 2 = selling, 3 = traditional products, 4 = medicinal plant, 5 = religious purpose, 6 = livestock fodder, 7 = others

Thank you for your answer!

Supplementary Questionnaire S2

Questionnaire
Interview with Male Farmer (or Female farmer in female headed household)

I would like to ask you some questions about use of plants and animals in the farm neighborhood. This is for a study to find out how to help farmers grow beneficial plants but are also useful to manage insects.
The objectives of this interview are:
To know what plants are currently used in the farmer field and surrounding agricultural areas.
To know what other benefits are derived from the farm neighborhood.
This interview may take 30 mins-1 hour. The data will be used for research purposes only. Will you consent to be interviewed?

Consent of respondent	Yes □1 No □2
Name of Interviewer:	
Date of interview	

II. Farmer information

10. What is famer name? ____________________Age_________
11. Address: ____________/__________/____________/___________
    Province: a. Prey Veng, b. Takeo
    District: a. Paem Ror, b. Preah Sdach, c. Bati, d. Tran
    Commune: a. Prey Kandeing, b. Lvear, c. Put Sar, d. Sam Bour
    Village: a. Sdao, b. Thom, c. Kandaul, d. Ro Vieng
12. What is your role in the family? Husband □1
    Head of household □2
    Child □3
    Extended family □4

4 Is rice farming your main occupation? yes □1 No □2

5 Aside from rice farming, what are your other income generating activities?
____

6 Are you active or significantly involved in farming? Yes □1 No □2

III. Farmer Situation

7 What crops do you grow, in what time of the year, and for what purpose?

Crops	Jan	Feb	Mar	Apr	May	Jun	Jul	Aug	Sep	Oct	Nov	Dec	Purpose	Code
														1= Eating<br>2= Sell<br>3=Traditional products<br>4= Medicinal plant<br>5=Religious purpose<br>6=Livestock fodder<br>7= Others

8 After harvest of rice, do you grow vegetables? Yes □1 No □2

9 Do you grow vegetables in a separate plot while growing rice? Yes □1 No □2

9.1 Where do you plant vegetables? Near the rice field□1

Near the house □2
In other plot far from rice field□3
Bunds □4

IV. Making use of plants and animals in the agriculture area

10 What plants growing in the area, which you collect or use, but do not plant yourself? Why do you collect these?

No	Name	Purpose	When and How many times you collect

Code: 1 = household consumption, 2 = selling, 3 = traditional products, 4 = medicinal plant, 5 = religious purpose, 6 = livestock fodder, 7 = others

15.1. Of the plants you identified can you name the 5 most important?

11 What animals do farmers collect from the farm and surrounding area? Why do you collect these?

No	Name	Purpose	When and How many times you collect

Code: 1 = household consumption, 2 = selling, 3 = traditional products, 4 = medicinal plant, 5 = religious purpose, 6 = livestock fodder, 7 = others

V. Experience in pest management

12 Have you heard of planting vegetables on the bunds? Yes □1 No □2

13 Have you planted vegetables on your bunds? Yes □1 No □2

27.1 If YES: Why did you plant vegetables on your bunds?
Food source □1 Income source□2 Pest management□3
Others□4 (explain) ____

27.1.1 What vegetables did you plant?______________________

27.1.2 What did you do to manage pests on these vegetables?
Insecticide-free□1 Insecticide□2

27.2 If NO: Would you consider planting vegetables on your bunds?

Yes □1 No □2
Please explain your answer: _____________________________________

14 Have you heard of planting flowers on the bunds? Yes □1 No □2

15 Have you planted flowers on your bunds? Yes □1 No □2

29.1 If YES: Why did you plant flowers on your bunds?
Pest management□1
Others □2 (explain) ____

25.1.1 What flowers did you plant? ________________________________

29.2 If No: Would you consider planting flowers on your bunds?

Yes □1 No □2

Please explain your answer (why/why not?):________________________________

30 Do you allow weeds/wild flowers to grow on your bunds for beneficial purposes?

Yes □1 No □2

30.1 If YES: Why did you allow weeds/wild flowers on your bunds?
Pest management□1
Others (explain) □2 ____

30.2 What weeds/wild flowers did you plant? _______________________________

30.3 If No: Would you consider planting weeds/wild flowers on your bunds?

Yes □1 No □2

Please explain your answer (why/why not?):

____

____

Thank you for your answer!

Supplementary Table S1. Arthropod communities collected during dry (DS) and wet (WS) season in ecological engineered (EE), conventional farmed (CR) and control fields with sweep netting.

Taxa	Functional group	DS		WS			Total
		CR	EE	Control	CR	EE	
Acari							
Gamasina	predator	65	127	60	59	178	489
Araneae							
Araneidae	predator	252	380	357	433	397	1819
Clubionidae	predator	1	1				2
Lycosidae	predator	8	11	34	7	38	98
Oxyopidae	predator	2	1		1	3	7
Salticidae	predator	1	3	7	9	20	40
Tetragnathidae	predator	108	189	187	210	205	899
Thomisidae	predator	2	3	7	6	2	20
Blattodea							
Blattodea	detritivore			1	1		2
Coleoptera							
Anthicidae	predator	1		5		1	7
Carabidae	predator	3	1	6	11	7	28
Chrysomelidae	herbivore	8	6	5	6	4	29
Coccinellidae	predator	71	53	65	97	56	342
Corylophidae	detritivore	7	5	3	3	8	26
Curculionidae	herbivore	1	3	1			5
Dytiscidae	predator		1				1
Elateridae	herbivore		1	1			2
Hydrophilidae	predator		1				1
Scarabaeidae	herbivore		1				1
Staphylinidae	predator	4	3	13	30	3	53
Tenebrionidae	herbivore	1	2				3
Collembola							
Entomobryidae	detritivore	22	262	63	9	221	577
Sminthuridae	detritivore			1	8		9
Dermaptera							
Dermaptera	predator			2	2	3	7
Diptera							
Brachycera	herbivore		2				2
Ceratopogonidae	herbivore	50	54	28	22	52	206
Chironomidae	detritivore	3429	4703	2522	2415	3457	16526
Chloropidae	herbivore	21	85	27	28	29	190
Culicidae	detritivore		6				6
Dolichopodidae	predator	1	2				3
Empididae	predator				1		1
Ephydridae	herbivore	48	73	50	84	47	302
Ochthera sauteri	predator	2					2
Muscidae	herbivore	3	4	10	11	17	45
Phoridae	detritivore	1	1	8	1	14	25
Pipunculidae	parasitoid	17	32	108	18	18	193
Platystomatidae	herbivore		15	1	1		17
Psilidae	herbivore			1	1		2
Sciomyzidae	predator		5				5

Syrphidae	pollinator	2				1	3
Tachinidae	parasitoid		1	3			4
Tipulidae	detritivore	2	5	16	16	20	59
Ephemeroptera							
Ephemeroptera	detritivore		3	24	26	63	116
Gastropoda							
Gastropoda	herbivore			48			48
Hemiptera							
Aleyrodidae	herbivore	495	216	232	223	265	1431
Alydidae	herbivore	6	2	4	1		13
Aphididae	herbivore	9	64	1	1	10	85
Cicadellidae	herbivore	301	438	325	512	325	1901
Coreidae	herbivore			2		1	3
Delphacidae	herbivore	235	204	160	698	452	1749
Nilaparvata lugens	herbivore	9	7	3	3	3	25
Sogatella furcifera	herbivore	3	22	25	10	45	105
Dictyopharidae	herbivore			1			1
Heteroptera	herbivore	115	22			2	139
Lygaeidae	herbivore	5	12	43	98	33	191
Meenoplidae	herbivore	1	2		1	3	7
Miridae	herbivore	4	8	7	12	3	34
Cyrtorhinus lividipennis	predator	16	24	285	169	296	790
Nabidae	predator				1		1
Naucoridae	predator					1	1
Pentatomidae	herbivore	8		3	4	3	18
Reduviidae	predator	1			5	2	8
Tingidae	herbivore		1				1
Veliidae	predator				1		1
Hymenoptera							
Aphelinidae	parasitoid	13	6				19
Bethylidae	parasitoid		1				1
Braconidae	parasitoid	38	39	48	42	30	197
Ceraphronidae	parasitoid	19	16	29	21	15	100
Chalcididae	parasitoid		6	2	3	3	14
Diapriidae	parasitoid	1			2	2	5
Dryinidae	parasitoid			15	5	2	22
Elasmidae	parasitoid	3	7	11	5	8	34
Encyrtidae	parasitoid	10	13	42	30	42	137
Eulophidae	parasitoid	39	119	50	42	40	290
Eupelmidae	parasitoid	1					1
Eurytomidae	parasitoid	2	4	2	2	4	14
Figitidae	parasitoid	2	3	1	4		10
Formicidae	predator	5	5	7	3	14	34
Ichneumonidae	parasitoid	23	41	47	60	29	200
Megaspilidae	parasitoid					1	1
Mymaridae	parasitoid	35	56	41	37	30	199
Platygastridae	parasitoid	14	21	81	51	86	253
Pteromalidae	parasitoid	29	31	39	24	27	150
Scelionidae	parasitoid	56	64	100	59	59	338
Trichogrammatidae	parasitoid	574	789	3002	1191	2607	8163

Vespidae	predator		4				4
Lepidoptera							
Crambidae	herbivore			1	1	1	3
Hesperiidae	herbivore			1		1	2
Lepidoptera larva	herbivore	15	17	27	36	28	123
Lycaenidae	herbivore					1	1
Pyralidae	herbivore	13	64	2	2	8	89
Mantodea							
Mantodea	predator	1					1
Odonata							
Coenagrionidae	predator	126	241	270	372	352	1361
Libellulidae	predator	1	3	5	20	7	36
Orthoptera							
Acrididae	herbivore	1	46	51	35	81	214
Gryllidae	herbivore		4	6	6		16
Pyrgomorphidae	herbivore	1	3		2	2	8
Tetrigidae	herbivore	2					2
Tettigoniidae	predator	14	34	10		3	61
Psocoptera							
Psocoptera	detritivore			5	3	2	10
Strepsiptera							
Strepsiptera	parasitoid	6	5			2	13
Thysanoptera							
Thripidae	herbivore	667	494	2320	861	1110	5452

Supplementary Table S2. Arthropods on bund crops (mung bean, sesame and sponge gourde) were sampled by walking transects on bunds using sweep nets. Collected arthropods were assigned to the function they have in rice fields.

Order	Family	Functional group	Sweep net
Acari	Gamasina	predator	30
Araneae	Araneidae	predator	206
Araneae	Oxyopidae	predator	2
Araneae	Salticidae	predator	2
Araneae	Tetragnathidae	predator	21
Araneae	Thomisidae	predator	2
Coleoptera	Anthicidae	predator	2
Coleoptera	Carabidae	predator	1
Coleoptera	Chrysomelidae	herbivore	15
Coleoptera	Coccinellidae	predator	37
Coleoptera	Corylophidae	detritivore	6
Coleoptera	Curculionidae	herbivore	4
Coleoptera	Dytiscidae	predator	1
Coleoptera	Scarabaeidae	herbivore	1
Coleoptera	Staphylinidae	predator	1
Coleoptera	Tenebrionidae	herbivore	1
Collembola	Entomobryidae	detritivore	14
Dermaptera		predator	1
Diptera	Calliphoridae	pollinator	1
Diptera	Ceratopogonidae	herbivore	16
Diptera	Chironomidae	detritivore	990
Diptera	Chloropidae	herbivore	40
Diptera	Dolichopodidae	predator	6
Diptera	Empididae	predator	1
Diptera	Ephydridae	herbivore	60
Diptera	Muscidae	herbivore	16
Diptera	Phoridae	detritivore	1
Diptera	Pipunculidae	parasitoid	4
Diptera	Platystomatidae	herbivore	5
Diptera	Syrphidae	pollinator	1
Diptera	Tachinidae	parasitoid	1
Ephemeroptera		detritivore	2
Hemiptera	Aleyrodidae	herbivore	10
Hemiptera	Aphididae	herbivore	440
Hemiptera	Cicadellidae	herbivore	108
Hemiptera	Delphacidae	herbivore	45
Hemiptera	Lygaeidae	herbivore	13
Hemiptera	Meenoplidae	herbivore	1
Hemiptera	Miridae	herbivore	6
Hemiptera	Miridae: *Cyrtorhinus lividipennis*	predator	57
Hemiptera	Pentatomidae	herbivore	6
Hemiptera	Reduviidae	predator	2
Hymenoptera	Aphelinidae	parasitoid	7
Hymenoptera	Apoidae	pollinator	3
Hymenoptera	Braconidae	parasitoid	4
Hymenoptera	Ceraphronidae	parasitoid	4

Hymenoptera	Chalcididae	parasitoid	1
Hymenoptera	Diapriidae	parasitoid	1
Hymenoptera	Dryinidae	parasitoid	1
Hymenoptera	Elasmidae	parasitoid	1
Hymenoptera	Encyrtidae	parasitoid	6
Hymenoptera	Eulophidae	parasitoid	67
Hymenoptera	Eurytomidae	parasitoid	3
Hymenoptera	Figitidae	parasitoid	1
Hymenoptera	Formicidae	predator	39
Hymenoptera	Halictidae	pollinator	1
Hymenoptera	Ichneumonidae	parasitoid	2
Hymenoptera	Mymaridae	parasitoid	20
Hymenoptera	Platygastridae	parasitoid	6
Hymenoptera	Pteromalidae	parasitoid	3
Hymenoptera	Scelionidae	parasitoid	26
Hymenoptera	Trichogrammatidae	parasitoid	163
Hymenoptera	Vespidae	predator	1
Isoptera	Isoptera	predator	1
Lepidoptera	Hesperiidae	herbivore	1
Lepidoptera	Lycaenidae	herbivore	2
Lepidoptera	Pyralidae	herbivore	16
Mantodea	Mantodea	predator	1
Odonata	Coenagrionidae	predator	19
Orthoptera	Acrididae	herbivore	20
Orthoptera	Gryllidae	herbivore	2
Orthoptera	Pyrgomorphidae	herbivore	8
Orthoptera	Tetrigidae	herbivore	1
Orthoptera	Tettigoniidae	predator	1
Thysanoptera	Thripidae	herbivore	342

Supplementary Tabel S3. Pairwise comparison of the ten main families (Tukey method) based on our computed models of family abundance collected in different treatments (CR= conventional rice field, EE= ecological engineered field, control) during dry (DS) and wet season (WS).

Season	Comparison	Family	Estimate	SE	p Value
DS	CR - EE	Araneidae	-0.338	0.578	0.559
	CR - EE	Chironomidae	-0.307	0.571	0.591
	CR - EE	Cicadellidae	-0.454	0.574	0.429
	CR - EE	Coccinellidae	0.029	0.607	0.962
	CR - EE	Delphacidae	0.043	0.579	0.941
	CR - EE	Entomobryidae	-1.524	0.891	0.087
	CR - EE	Mymaridae	-0.639	0.721	0.376
	CR - EE	Tetragnathidae	-0.166	0.616	0.787
	CR - EE	Thripidae	0.089	0.606	0.884
	CR - EE	Trichogrammatidae	-0.274	0.575	0.634
WS	Control - CR	Araneidae	-0.192	0.418	0.890
	Control - EE	Araneidae	-0.126	0.419	0.951
	CR - EE	Araneidae	0.066	0.419	0.986
	Control - CR	Chironomidae	0.091	0.414	0.974
	Control - EE	Chironomidae	-0.294	0.415	0.758
	CR - EE	Chironomidae	-0.386	0.414	0.620
	Control - CR	Cicadellidae	-0.288	0.423	0.775
	Control - EE	Cicadellidae	-0.051	0.422	0.992
	CR - EE	Cicadellidae	0.237	0.426	0.843
	Control - CR	Coccinellidae	-0.420	0.444	0.611
	Control - EE	Coccinellidae	-0.053	0.476	0.993
	CR - EE	Coccinellidae	0.368	0.471	0.715
	Control - CR	Delphacidae	-1.254	0.423	0.008
	Control - EE	Delphacidae	-0.912	0.421	0.078
	CR - EE	Delphacidae	0.343	0.419	0.692
	Control - CR	Entomobryidae	2.310	0.679	0.002
	Control - EE	Entomobryidae	-0.576	0.582	0.584
	CR - EE	Entomobryidae	-2.886	0.584	0.000
	Control - CR	Mymaridae	0.166	0.473	0.935
	Control - EE	Mymaridae	0.342	0.479	0.755
	CR - EE	Mymaridae	0.177	0.482	0.929
	Control - CR	Tetragnathidae	-0.070	0.425	0.985
	Control - EE	Tetragnathidae	-0.116	0.426	0.960
	CR - EE	Tetragnathidae	-0.045	0.424	0.994
	Control - CR	Thripidae	0.997	0.415	0.043
	Control - EE	Thripidae	0.791	0.415	0.136
	CR - EE	Thripidae	-0.206	0.415	0.873
	Control - CR	Trichogrammatidae	0.923	0.414	0.062
	Control - EE	Trichogrammatidae	0.106	0.414	0.965
	CR - EE	Trichogrammatidae	-0.817	0.414	0.052

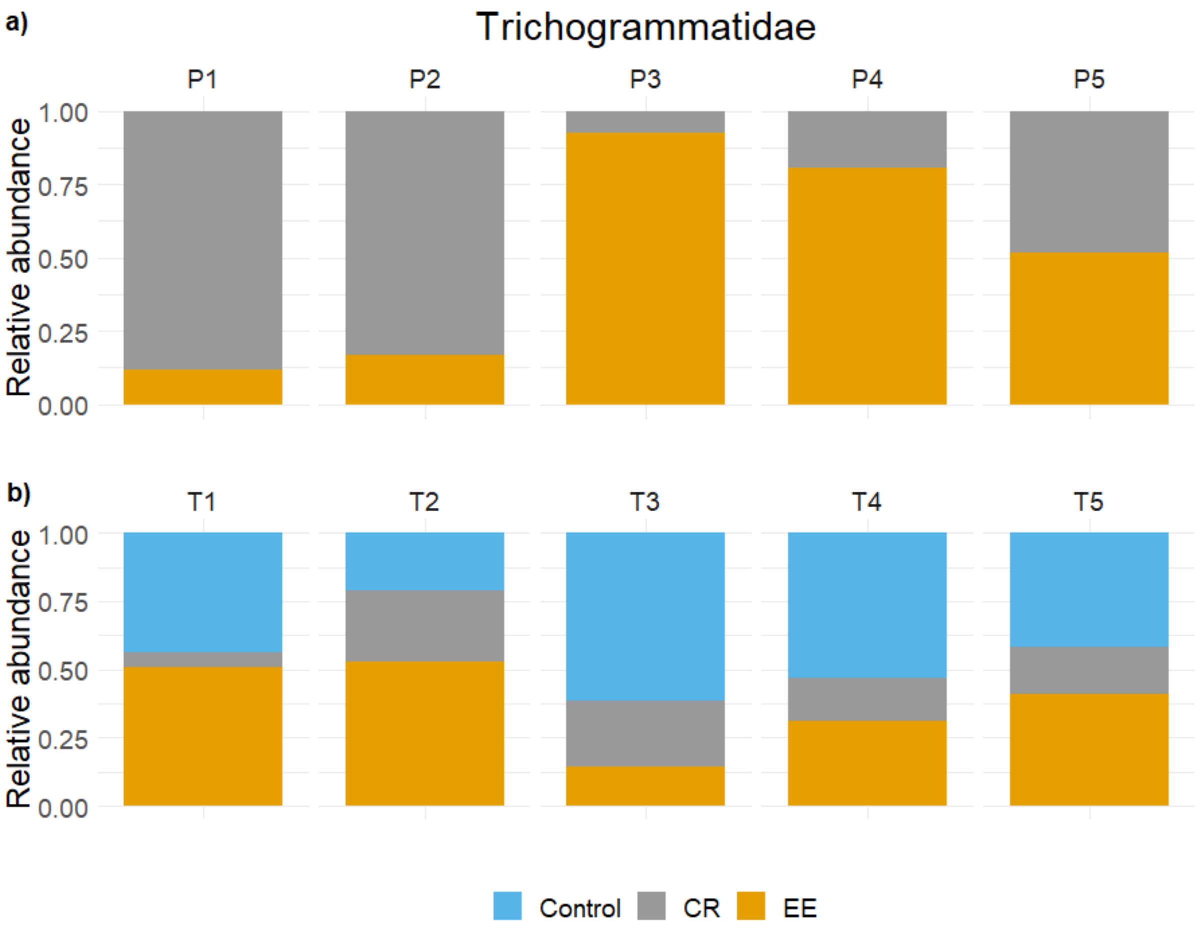

Supplementary Figure S1. Relative abundance of Trichogrammatidae (functional group of parasitoids) collected during a) dry and b) wet season in control, ecologically engineered (EE) and conventionally farmed rice fields (CR).

Supplementary Table S4. Comparison of family richness between the rice field pairs (P1-P5) and trios (T1-T5) collected during dry (DS) and wet (WS) season in conventionally treated fields (CR), ecologically engineered fields (EE) and control fields (Control).

Season	Region	Pairs	Treatment	Family richness
DS	Battambang	P1	CR	29
			EE	40
		P2	CR	26
			EE	45
	Kampong Thom	P3	CR	39
			EE	62
	Prey Veng	P4	CR	39
			EE	53
		P5	CR	46
			EE	56
WS	Prey Veng	T1	Control	44
			CR	41
			EE	51
		T2	Control	39
			CR	50
			EE	46
		T3	Control	50
			CR	41
			EE	45
		T4	Control	53
			CR	51
			EE	43
		T5	Control	45
			CR	38
			EE	36
